# Combination of Voluntary Wheel Running and Oral Intake of Lactate Improves Object and Spatial Recognition Memory in Association With Hippocampal Insulin‐Like Growth Factor‐1 in Mice

**DOI:** 10.1002/fsn3.72091

**Published:** 2026-07-06

**Authors:** Momoka Ota, Nana Esaki, Kai Sahara, Toshiro Matsui, Takanori Tsuda

**Affiliations:** ^1^ School of Bioscience and Biotechnology and Graduate School of Bioscience and Biotechnology Chubu University Kasugai Aichi Japan; ^2^ Department of Bioscience and Biotechnology, Faculty of Agriculture Graduate School of Kyushu University Fukuoka Japan

**Keywords:** cognitive improvement, exercise, hippocampus, insulin‐like growth factor‐1, lactate

## Abstract

Lactate is a food component that is sometimes also used as a food additive. Recent research has reported that lactate may be a crucial signaling molecule and energy substrate. Furthermore, lactate has been suggested to play a crucial role in learning and memory in the brain. It has been reported that high‐intensity exercise improves cognitive function via an increase in the blood lactate concentration. However, most studies have focused on the intraperitoneal administration of lactate or on endogenous lactate elevation via high‐intensity exercise, rather than on oral intake. Rather than exercise or lactate alone, the combination of the two might further enhance cognitive function. After confirming that 50 mM of lactate (L50) intake alone did not improve cognitive function, we demonstrated that the combination of voluntary exercise and L50 for 4 weeks significantly improved learning and memory in mice, despite the fact that exercise alone did not (*p* < 0.05). Mechanistically, this cognitive boost was associated with enhanced protein expression of synaptic plasticity markers, a significant increase in hippocampal neurogenesis, and a significant elevation in insulin‐like growth factor‐1 protein levels, along with activation of calcium calmodulin‐dependent protein kinase II in the hippocampus. This effect was notably independent of changes in brain‐derived neurotrophic factor expression levels. These results indicate that lactate might be beneficial as a dietary supplement during exercise that enhances learning and memory in both young and older people.

## Graphical abstract

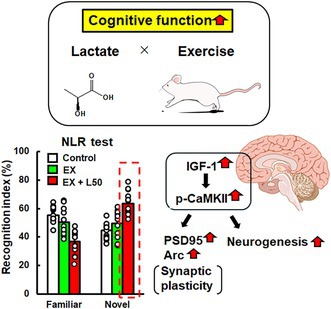

**Abbreviations**
Arc: activity‐regulated cytoskeletal‐associated protein
BDNF: brain‐derived neurotrophic factor
BrdU: 5‐bromo‐2′‐deoxyuridine
CaMKII: calcium calmodulin‐dependent protein kinase II
ERK: extracellular signal‐regulated kinase
FGF‐2: fibroblast growth factor‐2
GAPDH: glyceraldehyde‐3‐phosphate dehydrogenase
IGF‐1: insulin‐like growth factor‐1
IGF‐1Rβ: insulin‐like growth factor‐1 receptor β
NeuN: neuronal nuclei
NLR: novel location‐recognition
NOR: novel object‐recognition
PSD95: postsynaptic density protein 95
VEGF: vascular endothelial growth factor

## 1 Introduction

Lactate is used as a component of many food ingredients as well as a food additive. Lactate is produced by the body during exercise but was previously thought to be an unnecessary metabolite. However, lactate passes through the blood–brain barrier (Knudsen et al. 1991) and is known to be an important energy source in the brain as well as the peripheral tissues (van Hall et al. 2009). Furthermore, lactate functions as a signaling molecule in a number of tissues (Brooks 2018). In particular, lactate has been suggested to play a crucial role in brain function, especially in cognitive processes. For example, animal studies have shown that lactate is involved in learning and memory in the brain (Lev‐Vachnish et al. 2019), and the inhibition of lactate transport to the hippocampus impairs memory formation in mice (Suzuki et al. 2011). In vitro experiments using primary hippocampal cells (Hu et al. 2021) and in vivo studies in mice (El Hayek et al. 2019) have demonstrated that the administration of lactate increased the expression of brain‐derived neurotrophic factor (BDNF), a crucial protein associated with learning and memory. Furthermore, lactate restores neurogenesis and decreases depressive behavior in mice (Carrard et al. 2021). Taken together, these results highlight the involvement of lactate in cognitive function in the brain. However, most studies focused on the intraperitoneal or intramuscular administration of lactate or the endogenous elevation in lactate caused by high‐intensity exercise, not on oral intake.

In clinical and epidemiological studies, regular exercise has numerous benefits, including increased energy expenditure (Jakicic 2003) and improved insulin sensitivity via increased glucose and lipid metabolism (Goodyear and Kahn 1998), and it contributes to the improvement or prevention of cardiovascular disease (Joyner and Green 2009; Lavie et al. 2015). Among the health benefits of exercise, the relationship of exercise with cognitive function is particularly notable, and the World Health Organization recommends at least 150 min of moderate exercise per week to reduce the risk of cognitive decline (World Health Organization 2019). Clinical research has shown that regular exercise is effective in suppressing cognitive decline in patients with Alzheimer's disease as well as elderly individuals (Morris et al. 2017; Yu et al. 2021; Thomas et al. 2020; Erickson et al. 2011). With respect to the relationship between lactate and exercise, it has been reported that acute high‐intensity interval exercise improves cognitive function via an increase in the concentration of blood lactate (Rodriguez et al. 2018). In addition, in human subjects, the administration of lactate has been reported to increase blood BDNF concentrations in sedentary young adults (Schiffer et al. 2011). Furthermore, acute high‐intensity interval training or sodium lactate administration has been shown to promote cerebral vascular endothelial growth factor (VEGF) expression and angiogenesis, which are related to cognitive function (Morland et al. 2017). These findings suggest that lactate is an important signaling molecule that mediates the cognitive‐enhancing effects of exercise.

Regarding exercise intensity, it has been reported that the risk of developing dementia is lower in older adults who engage in moderate‐ to high‐intensity exercise than in those who engage in low‐intensity exercise (e.g., light walking) (Laurin et al. 2001; Cassilhas et al. 2007; Ihira et al. 2022). However, not all people are able to exercise continuously above a certain intensity. Therefore, rather than aiming for the highly beneficial effects through exercise or lactate alone, improved cognitive function might be achieved through the combination of lactate intake and exercise.

Based on this background, we hypothesized that the oral intake of lactate combined with exercise would significantly improve cognitive function compared to either intervention alone. In this study, “exercise” was specifically defined as voluntary wheel running, and “cognitive function” focused on object and spatial recognition memory as assessed by the novel location‐recognition (NLR) and novel object‐recognition (NOR) tests. We investigated the effects of this combined intervention on cognitive performance, specifically object and spatial recognition memory, in mice. Furthermore, we analyzed the expression of key molecular markers in the hippocampus to elucidate the underlying mechanisms that mediate these effects.

## 2 Materials and Methods

### 2.1 Chemicals and Antibodies

Sodium lactate and 5‐bromo‐2′‐deoxyuridine (BrdU) were obtained from Fujifilm Wako Pure Chemical Corporation (Osaka, Japan). A complete list of antibodies used in this study is presented in Supporting Information: Materials and Methods.

### 2.2 Animal Experiments

The animal experiments in this study received approval from the Animal Experiment Committee at Chubu University. Furthermore, all procedures involving mice adhered to the applicable institutional guidelines for their care and use (permission nos 202110016 and 202410018). The experimental procedure consisted of two stages. First, the dose–response effect of lactate alone was established (Phase 1). Subsequently, the combined effect of a sub‐threshold dose of lactate (L50) and exercise was evaluated (Phase 2). In Phase 2, the L50‐only group was omitted to minimize animal usage in accordance with the 3Rs principle, as its independent effect had already been statistically confirmed as non‐significant in Phase 1.

### 2.3 Dosage Information

The control group received water, while the lactate group was provided with a sodium lactate solution (either 50 or 100 mM) as their drinking water, with both solutions being replaced every other day. The lactate concentration in the drinking water was based on our previous research (Esaki et al. 2023), which demonstrated that oral intake of 100 mM sodium lactate solution effectively induces physiological changes, such as the browning of white adipose tissue, without affecting food consumption or glucose tolerance in mice (Esaki et al. 2023). The average daily intake of sodium lactate was calculated to be around 1–2 g/kg of body weight, based on the daily water consumption during the study period. While earlier studies using intraperitoneal administration of 2.0–3.0 g of sodium lactate per kilogram of body weight have reported metabolic changes in the brain and skeletal muscle (Lezi et al. 2013; Hoshino et al. 2014), the dose used in the present study was primarily selected to provide a sufficient yet safe physiological stimulus through a practical oral route, consistent with our previous findings (Esaki et al. 2023).

### 2.4 Effects of Lactate Intake Without Exercise on Cognitive Function

Seven‐week‐old male ICR mice (Japan SLC, Hamamatsu, Japan) were kept in a controlled animal facility with a 12‐h light/dark schedule, with lights on from 08:00 to 20:00, and a constant temperature of 23°C ± 3°C. After a week of acclimatization, the mice were randomly divided into three groups: control, L50, and L100. The control group received water, while the L50 and L100 groups were provided with sodium lactate solution (50 and 100 mM, respectively) as their drinking water. Throughout the study, the mice had unrestricted access to a semi‐purified standard diet, which was a modified version of the AIN‐93G diet (Nishikawa et al. 2019; Reeves et al. 1993). The duration of the experimental intervention was 28 days. In the final week of the experiment, behavioral tests were conducted to evaluate cognitive function, as outlined in Sections 2.6 and 2.7. Upon completion of the experiments, the mice were anesthetized using isoflurane. Blood samples were drawn using a syringe containing heparin, and plasma was separated by centrifugation. The hippocampus was extracted and promptly frozen in liquid nitrogen and stored at −80°C until needed. For protein detection, tissue samples were homogenized and analyzed via immunoblotting using glyceraldehyde‐3‐phosphate dehydrogenase as a loading control, as described in our previous research (Kojima et al. 2021; Kato and Tsuda 2023; Suzuki et al. 2025). The details of the western blot analysis procedure are presented in the Supporting Information.

### 2.5 Effects of Lactate Combined With Voluntary Exercise on Cognitive Function

Seven‐week‐old male ICR mice (Japan SLC, Hamamatsu, Japan) were kept as described above. After a week of acclimatization, the mice were randomly divided into control, exercise, or exercise + L50 groups. In line with our previous research (Suzuki et al. 2025), the mice in both the exercise and exercise + L50 groups were kept in individual cages, where they had continuous access to a computer‐monitored inclined voluntary running wheel with an outer diameter of 15.5 cm (LCW‐M4; Melquest Ltd., Toyama, Japan) for the entirety of the experiment. The number of wheel rotations was tracked using a counting system (CNT‐10; Melquest Ltd.) positioned outside the cage, which enabled the running distance to be calculated for each mouse. The duration of the experimental intervention was 28 days. In the final week leading up to the conclusion of the experiment, behavioral assessments were conducted to evaluate cognitive function, as outlined below in Sections 2.6 and 2.7 (Suzuki et al. 2025). After the behavioral experiments were completed, plasma, hippocampus, liver, and skeletal muscle samples were obtained and analyzed as described in Section 2.4.

### 2.6 NLR Test

An NLR test was conducted as previously reported (Suzuki et al. 2025). The NLR test is a simple yet effective means of evaluating spatial memory that relies on the hippocampus (Ikeda et al. 2021; van Praag et al. 1999). Briefly, following the habituation sessions outlined in our previous research (5‐min sessions over 3 consecutive days) (Denninger et al. 2018), two identical objects were positioned along the same line within the arena, and the amount of time each mouse spent investigating these objects was recorded for 5 min per mouse. Testing sessions took place 24 h after the acquisition trials. During these sessions, one object was repositioned diagonally in the arena, and the amount of time each mouse spent exploring the object in the new location was recorded for 5 min per mouse. Both the acquisition and testing trials were recorded using a video camera mounted above the arena. The analysis was performed using a video tracking system (SMART 3.0; Panlab, S.L., Barcelona, Spain). To remove scent cues between trials, all objects were cleaned with 70% ethanol. Exploration time (s) was analyzed based on the amount of time spent at novel and familiar locations. Recognition indices (%) for both locations were calculated by dividing the time each mouse spent at a location by the total investigation time, indicating how thoroughly each mouse explored the novel location. The discrimination index for the novel location was determined using the formula: Discrimination index = (duration of novel location exploration—duration of familiar location exploration)/(total exploration time for both locations).

### 2.7 NOR Test

A NOR test was conducted as previously reported (Vogel‐Ciernia and Wood 2014; Denninger et al. 2018). Briefly, after the habituation sessions along with the NLR test, two identical items were positioned along the same line in the arena, and the amount of time it took for the mice to search for these items was recorded for 5 min per mouse. Testing sessions were conducted 24 h after the acquisition trials. In the test session, one of the objects in the arena was replaced by a novel object, and the amount of time it took for the mouse to explore the novel object was measured at 5 min per mouse. The acquisition and testing trials were recorded and analyzed as described above for the NLR test. Recognition indices (%) for both objects were calculated by dividing the time each mouse spent at an object by the total investigation time, indicating how thoroughly each mouse explored the novel object. The discrimination index for the novel object was determined using the formula: Discrimination index = (duration of novel object exploration − duration of familiar object exploration) / (total exploration time for both objects).

### 2.8 Measurement of the Plasma and Hippocampal Lactate Concentrations

The plasma lactate concentration was determined using a Lactate Assay Kit (MAK064; Sigma–Aldrich, St. Louis, MO), following the manufacturer's protocol. The lactate concentration in the hippocampus was determined according to the method of Ikeda et al. (Ikeda et al. 2021). Briefly, frozen tissues were homogenized with 1 M perchloric acid and centrifuged to remove proteins. The supernatants were adjusted to pH 7.0 using sodium hydroxide, and the lactate concentrations were measured using the assay kit described above.

### 2.9 Double Immunofluorescence Staining of BrdU and Neuronal Nuclei (NeuN)

ICR mice (control, exercise, and exercise + L50 groups) were maintained for 28 days as reported above. BrdU was intraperitoneally injected into mice (50 mg/kg) once per day from day 18 to 22. This timing was selected to label newborn cells during the stable intervention period. Following the established protocol for assessing the survival and early differentiation of newborn neurons (van Praag et al. 1999), the mice were sacrificed 6 days after the final BrdU injection. After 28 days, the brain was harvested and fixed in 4% paraformaldehyde at 4°C for 24 h. The tissues were embedded in paraffin and sectioned into 5‐μm‐thick slices for immunofluorescence staining and then deparaffinized and rehydrated using xylene, ethanol, and water as described in our previous reports (Nishikawa et al. 2019, 2016). Double staining of BrdU and NeuN was performed according to a previously reported protocol, with slight modifications (Liu et al. 2022). Briefly, 10 brain sections per mouse were used for every 1‐in‐7 in series. The sections were incubated with 1 M HCl at 37°C for 30 min, and subsequently incubated with 0.1 M sodium borate buffer (pH 8.5) and washed with phosphate‐buffered saline for 15 min. After blocking with blocking buffer (phosphate‐buffered saline containing 5% goat serum and 0.3% Triton X‐100) for 1 h at room temperature, the sections were incubated with anti‐BrdU (1:300) and anti‐NeuN (1:300) antibodies for 16 h at 4°C. They were subsequently reacted with Alexa Fluor 488 conjugated anti‐rabbit IgG (for NeuN) and Alexa Fluor 555 conjugated anti‐mouse IgG (for BrdU) for 90 min at room temperature. Autofluorescence quenching was performed using the Vector TrueVIEW Autofluorescence Quenching Kit (Vector Laboratories Inc., Newark, CA), following the manufacturer's instructions. Immunofluorescence double‐positive cells (BrdU^+^/NeuN^+^) were detected using a fluorescence microscope system (IX71; Olympus, Tokyo, Japan) with a microscope digital camera (DP74; Olympus). The number of BrdU^+^/NeuN^+^ double‐positive cells in the granule cell and subgranular layers of the dentate gyrus in each section was summed and the total number of the positive cells was multiplied by 7.

### 2.10 Statistical Analysis

**FIGURE 1:**
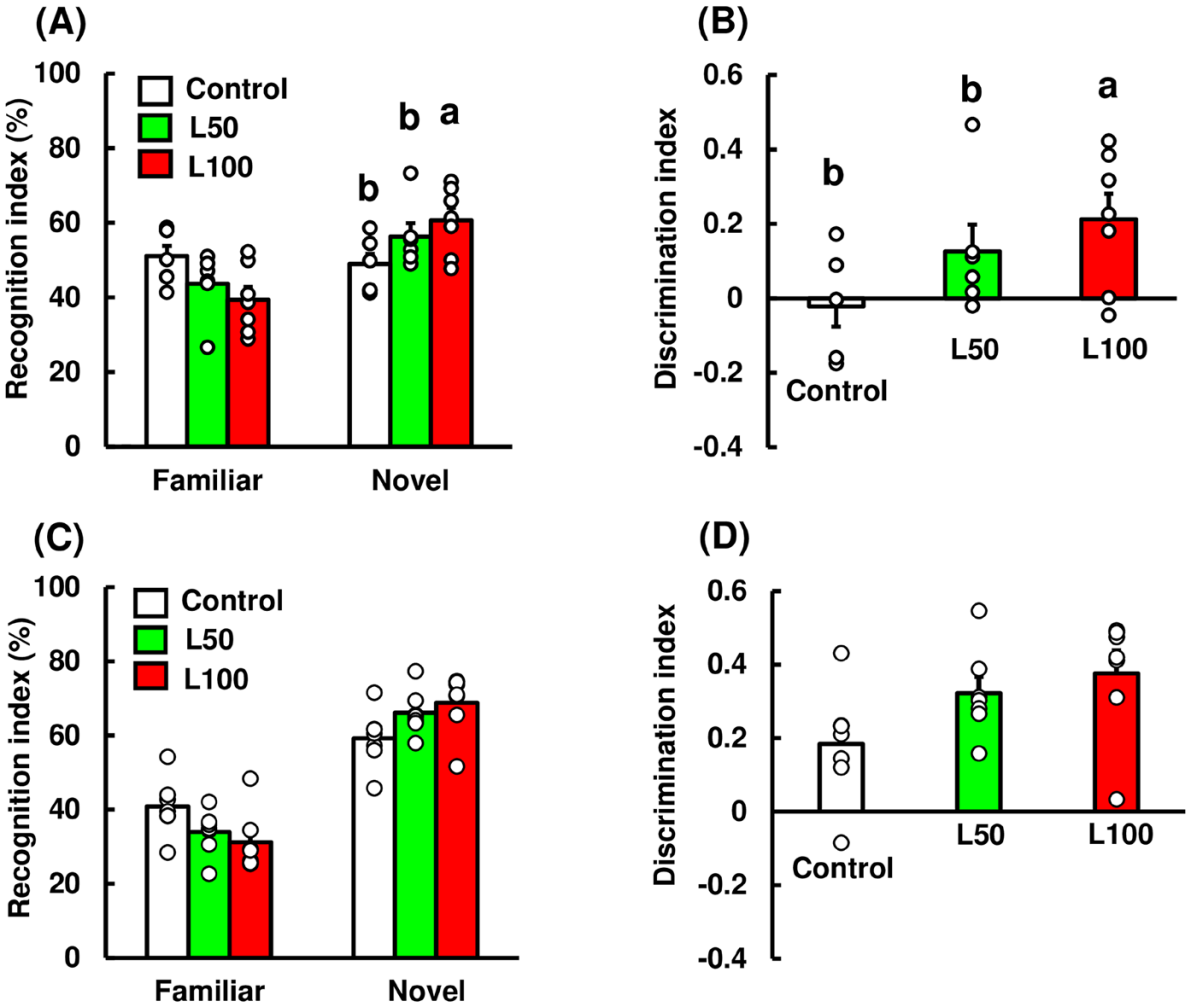
Recognition index (%) and discrimination index after the NLR test (A, B), and the NOR test (C, D) in the control, L50, and L100 groups. Data are shown as means ± SEM (*n* = 7). Statistical significance was determined by one‐way ANOVA followed by Tukey's post hoc test. Values with different letters are significantly different (*p* < 0.05). Cohen's *d* values for primary comparisons are reported in the Results section.

**FIGURE 2:**
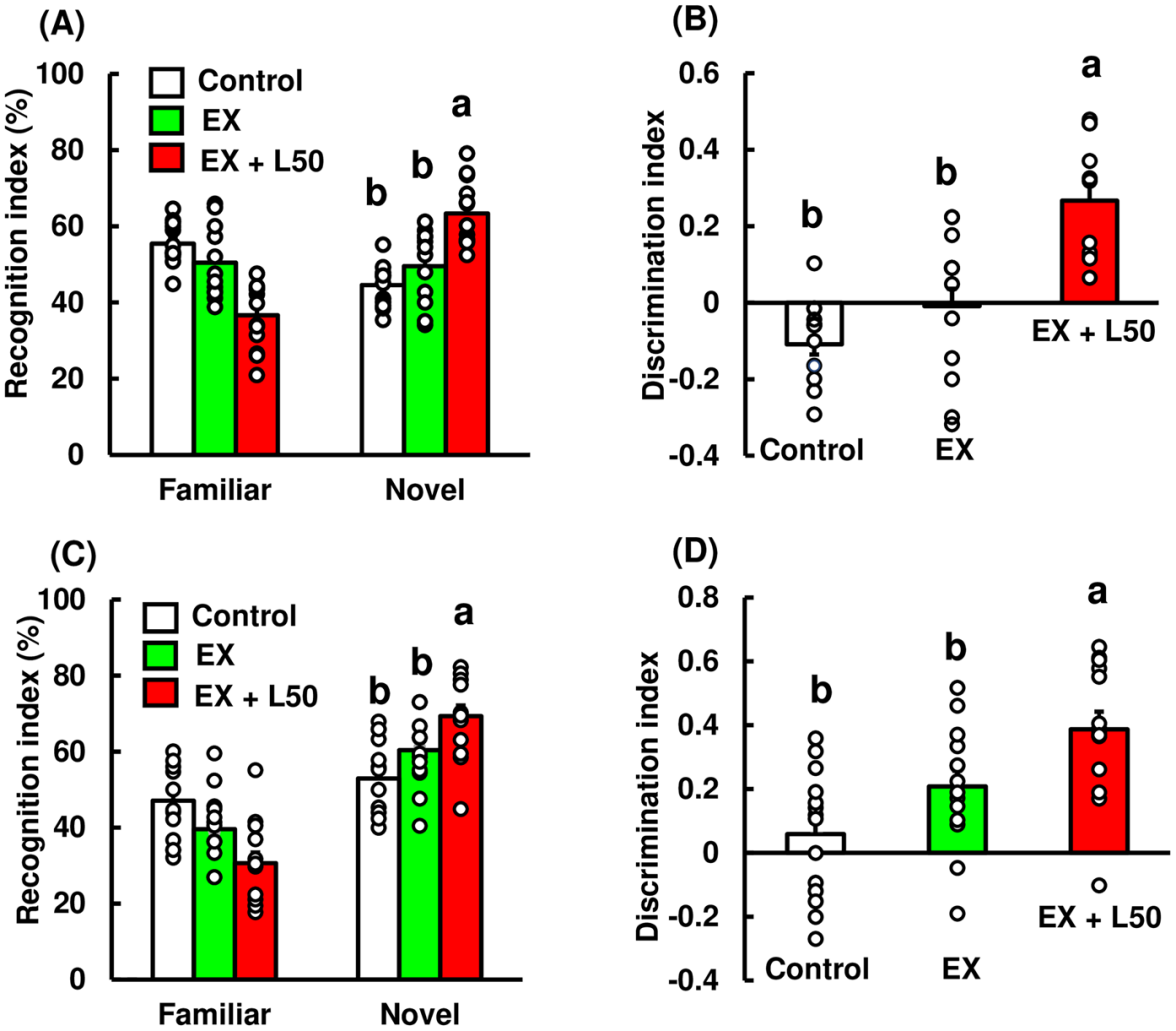
Recognition index (%) and discrimination index after the NLR test (A, B), and the NOR test (C, D) in the control, EX, and EX + L50 groups. Data are shown as means ± SEM (*n* = 14). Statistical significance was determined by one‐way ANOVA followed by Tukey's post hoc test. Values with different letters are significantly different (*p* < 0.05). Cohen's *d* values for primary comparisons are reported in the results section. EX, exercise.

The data were analyzed using one‐way ANOVA followed by Tukey's post hoc test for multiple comparisons. This conservative method was employed to ensure rigorous pairwise comparisons between experimental groups while controlling for the family‐wise error rate. All results are reported as means ± SEMs. Effect sizes (Cohen's *d*) were reported for the primary pairwise comparisons in Figures 1 and 2. A *p* < 0.05 was considered to indicate significance.

## 3 Results

### 3.1 Effects of Lactate Intake Without Exercise on Cognitive Function

We first examined whether 4 weeks of oral intake of lactate (L50 or L100) without exercise could improve cognitive function in mice. Final body weight, total food intake, and water intake did not differ among the three groups (Table S1). Tukey's post hoc test revealed that L100 significantly improved memory performance in the NLR test compared with the control group (Cohen's *d* = 1.43), whereas no significant differences were observed among the three groups in the NOR test (Figure 1). Additionally, L50 showed no significant difference from the control group (*p* > 0.05) in both tests (Figure 1). Based on these results, L50 was selected as the optimal sub‐threshold dose for investigating the potential potentiating effects of combined treatment with exercise.

Interestingly, despite the cognitive improvement observed in the L100 group, hippocampal BDNF protein levels were not significantly elevated (Figure S1).

### 3.2 L50 Combined With Exercise Significantly Improves Cognitive Function in Mice

The results presented above show that the L50 diet did not have a significant effect on cognitive function in mice. We next examined whether L50 combined with exercise for 4 weeks significantly improved cognitive function. Total food intake did not differ among the groups. Final body weight in both the exercise and exercise + L50 groups was significantly lower compared with the control group (Table S2). Total running distance did not differ between the exercise alone and the exercise + L50 groups (Table S2). The recognition index and discrimination index results from the NLR test were significantly greater in the exercise + L50 group than in the control group (Cohen's *d* = 2.65 and 2.74, respectively), although exercise alone had no effect on these indices (Figure 2A,B). Tukey's post hoc test also revealed that the exercise + L50 group showed significantly higher indices compared with the exercise‐alone group (Cohen's *d* = 1.56 for recognition index; *d* = 1.62 for discrimination index). Furthermore, similar to the results of the NOR test, both the recognition index and discrimination index were significantly greater in the exercise + L50 group compared with the control group (Cohen's *d* = 1.61 for both) (Figure 2C,D).

### 3.3 L50 Combined With Exercise Significantly Induces Neurogenesis in the Hippocampus in Mice

**FIGURE 3:**
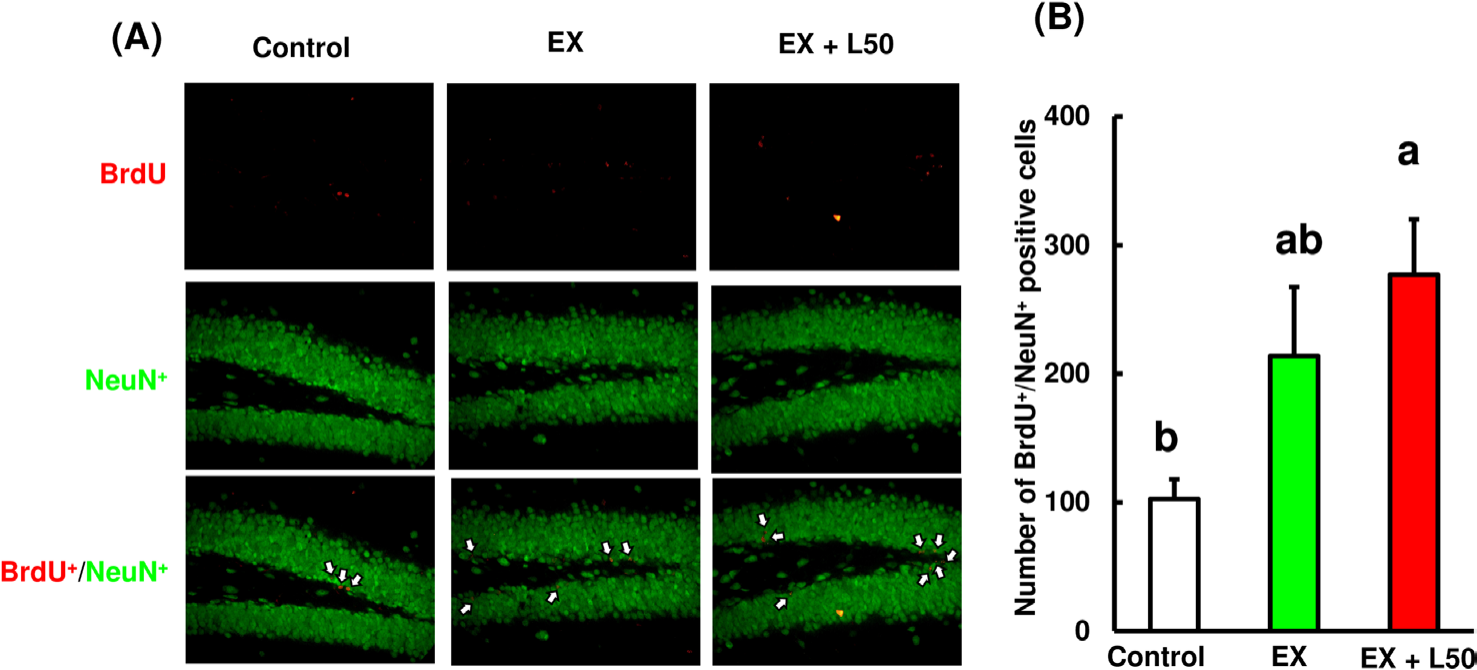
Representative immunofluorescence staining images of BrdU^+^, NeuN^+^, and merged images in the dentate gyrus of the control, EX, and EX + L50 groups (A). Numbers of BrdU^+^ and NeuN^+^ double‐positive cells in the dentate gyrus of the control, EX, and EX + L50 groups (B). Data are shown as means ± SEM (*n* = 3). Values with different letters are significantly different (*p* < 0.05), EX, exercise.

To determine whether the improved cognitive function resulting from combined exercise and L50 intake is associated with the promotion of neurogenesis, BrdU was administered to mice, and double immunofluorescence staining of NeuN (a reliable marker for mature neurons) and BrdU was performed in the dentate gyrus. Typical immunofluorescence detection of BrdU, NeuN, and merged images indicated that exercise and L50 group induced double‐positive staining of BrdU and NeuN (Figure 3A). The number of BrdU and NeuN double‐positive cells was significantly increased in the exercise and L50 group compared with the control (Figure 3B).

### 3.4 Effects of L50 Combined With Exercise on the Plasma and Hippocampal Lactate Concentration, and MCT2 Expression in the Hippocampus

**FIGURE 4:**
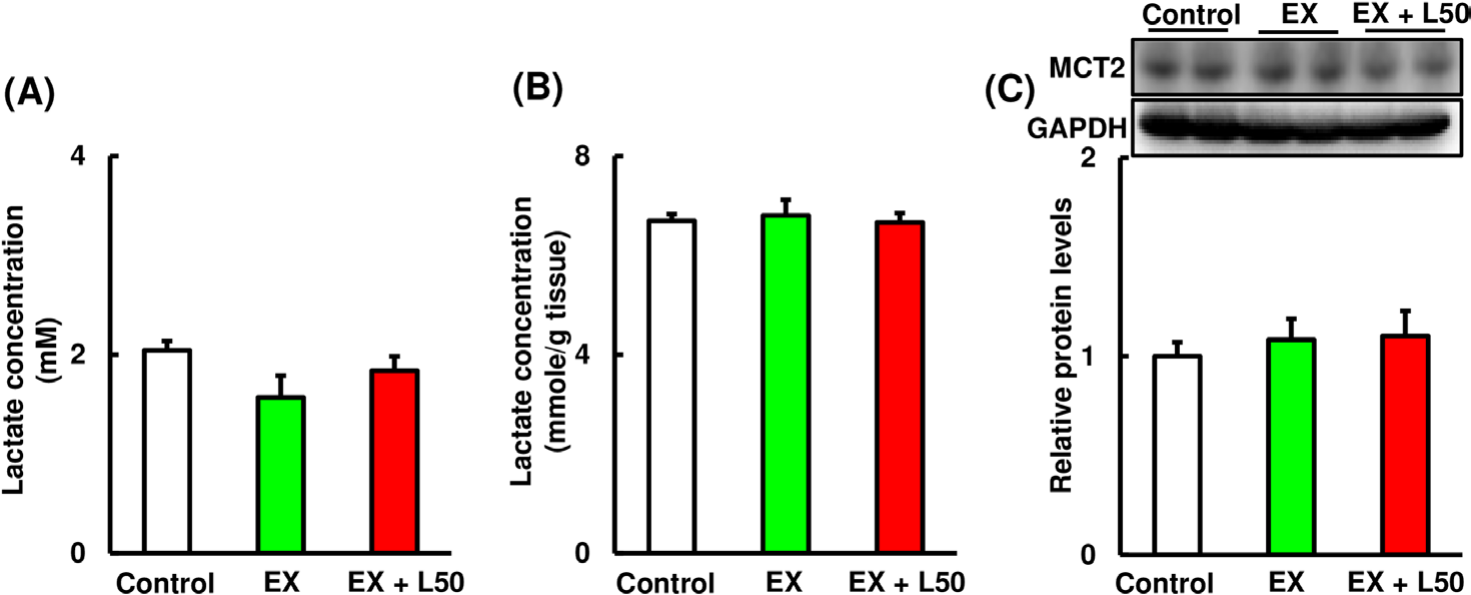
Plasma (A) and hippocampal (B) lactate concentration across 28 days in the control, EX, and EX + L50 groups. Expression levels of hippocampal proteins (MCT2 and GAPDH) in the control, EX, and EX + L50 groups (C). The MCT2 protein level is reported as fold‐changes compared with the control group (set at 1), following normalization to the GAPDH protein level. Data are shown as means ± SEM (*n* = 7). Values with different letters are significantly different (*p* < 0.05). EX, exercise; GAPDH, glyceraldehyde‐3‐phosphate dehydrogenase.

Neither the plasma nor hippocampal lactate concentrations differed significantly among the groups (Figure 4A,B). MCT2 is a crucial transporter for the influx of lactate in neurons, and blocking MCT2 inhibits neurogenesis and impairs lactate‐mediated cognitive improvement (Lev‐Vachnish et al. 2019). In addition, a previous study reported that exercise induced MCT2 expression in the brain (Takimoto and Hamada 2014). Although the expression levels of MCT2 proteins in the EX and EX+L50 groups showed a slight upward trend compared to the control, the differences did not reach statistical significance (Figure 4C).

### 3.5 L50 Combined With Exercise Significantly Increases the Expression of Markers of Neural and Synaptic Activity but Does Not Affect BDNF Levels in the Hippocampus of Mice

**FIGURE 5:**
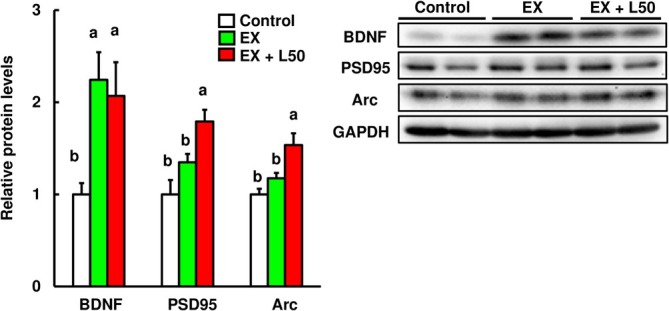
Expression levels of hippocampal proteins (BDNF, PSD95, Arc, and GAPDH) in the control, EX, and EX + L50 groups. Protein levels are reported as fold‐changes compared with the control group (set at 1), following normalization to the GAPDH protein level. Data are shown as means ± SEM (*n* = 7). Values with different letters are significantly different (*p* < 0.05). EX, exercise; GAPDH, glyceraldehyde‐3‐phosphate dehydrogenase.

Given the significant improvement in cognitive function in the exercise + L50 group, we wondered why the combination of L50 with exercise improved cognitive function while L50 or exercise alone did not. BDNF is a key protein in hippocampus‐associated learning and memory (Tyler et al. 2002). Contrary to our expectations, hippocampal BDNF protein levels in both the exercise alone and exercise + L50 groups were significantly higher than those in the control group (Figure 5). Both intervention groups showed a similar extent of increase in BDNF expression, and no additional elevation was observed in the combined treatment group (exercise + L50) compared to the exercise alone group. However, postsynaptic density protein 95 (PSD95), which is a plasticity‐related protein, and activity‐regulated cytoskeletal‐associated protein (Arc), which is a marker protein of neural and synaptic activity, were significantly greater in the exercise + L50 group (Figure 5).

### 3.6 Hippocampal IGF‐1 Levels Were Significantly Increased in the Exercise + L50 Group

**FIGURE 6:**
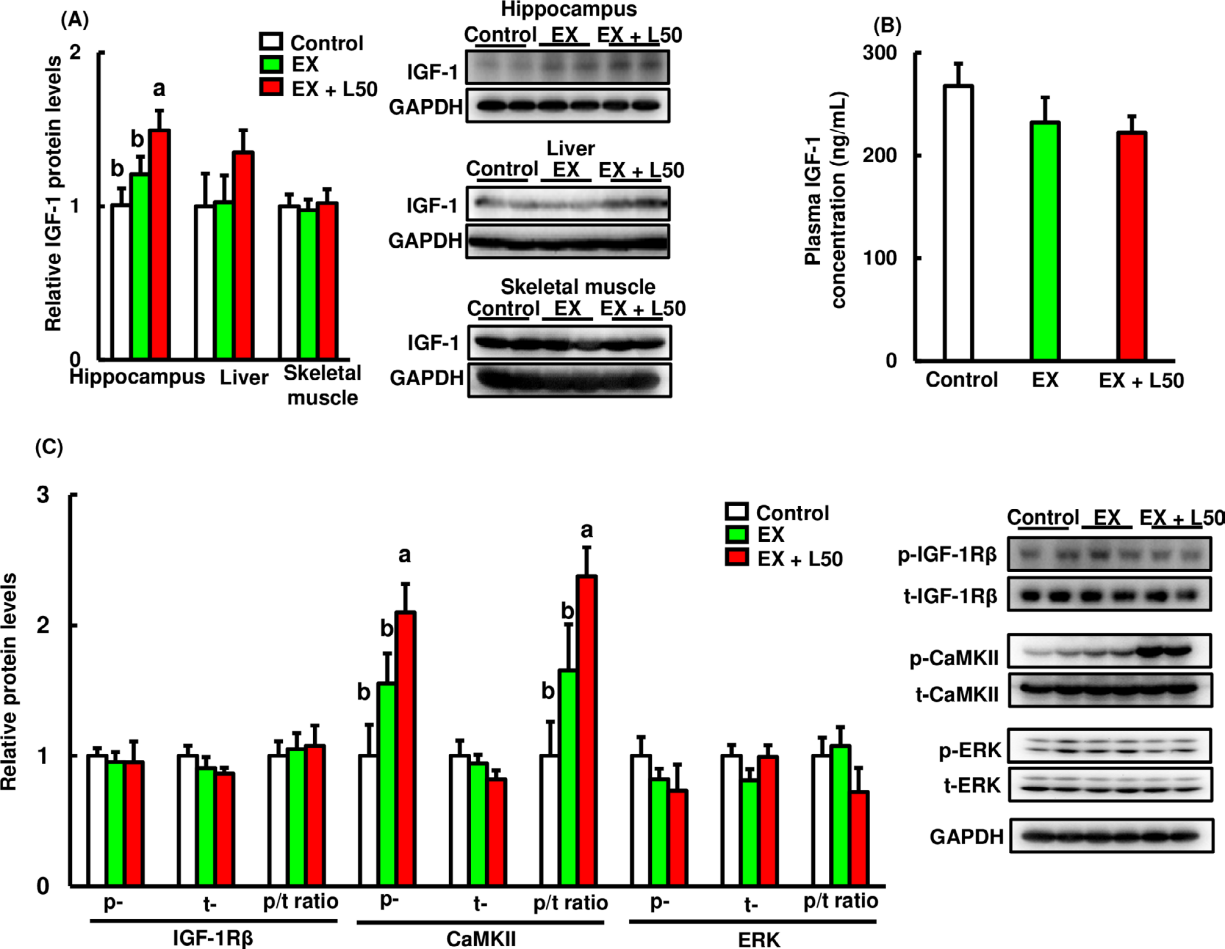
Protein expression levels of IGF‐1 in the hippocampus, liver, and skeletal muscle in the control, EX, and EX + L50 groups (A). Plasma IGF‐1 concentrations in the control, EX, and EX + L50 groups (B). Hippocampal protein expression levels of p‐IGF‐1Rβ, t‐IGF‐1Rβ, p/t ratio of IGF‐1β, p‐CaMKII, t‐CaMKII, p/t ratio of CaMKII, p‐ERK, t‐ERK, p/t ratio of ERK, and GAPDH in the control, EX, and EX + L50 groups. Protein levels are expressed as fold‐change relative to the control (= 1), following normalization to the GAPDH protein level (A, C). Data are presented as means ± SEM (*n* = 7). Values with different letters are significantly different (*p* < 0.05). EX, exercise; GAPDH, glyceraldehyde‐3‐phosphate dehydrogenase; p‐, phospho‐; t‐, total‐.

IGF‐1 is reported to mediate improvements in cognitive function in the hippocampus (Glasper et al. 2010; Farias Quipildor et al. 2019). In addition, exercise promotes neurogenesis and the regulation of learning and memory via increased IGF‐1 expression in the hippocampus or peripheral tissues (Trejo et al. 2008). Hippocampal expression of the IGF‐1 protein was significantly elevated in the exercise + L50 group, whereas exercise alone had no effect on its expression (Figure 6A). IGF‐1 is expressed mainly in the liver and skeletal muscle (Pharaoh et al. 2020; Matheny et al. 2009). As shown in Figure 6A, the protein level of IGF‐1 in both the liver and skeletal muscle did not differ among the groups. In addition, the plasma level of IGF‐1 did not significantly differ among the groups (Figure 6B).

A substantial increase in IGF‐1 expression in the hippocampus activates the downstream signaling pathways of IGF‐1, leading to improved learning and memory (Hu et al. 2016). Activation of CaMKII (phosphorylation at Thr286) is crucial for improving learning and memory abilities (Impey et al. 1998; Xia and Storm 2005; Moriguchi et al. 2014) and it is thought to be involved in IGF‐1‐mediated cognitive improvement (Hu et al. 2016). Therefore, we examined the expression levels of IGF‐1 receptor β (IGF‐1Rβ) and CaMKII proteins in the hippocampus. Although a marked degree of IGF‐1Rβ phosphorylation was not observed in the exercise + L50 group, the protein expression levels of phospho‐CaMKII and the phospho‐CaMKII/total CaMKII ratio were significantly elevated in the exercise + L50 group compared with the control group (Figure 6C). Extracellular signal‐regulated kinase (ERK) is also thought to be involved in improving learning and memory in the hippocampus (Impey et al. 1998; Xia and Storm 2005), and it has been reported that ERK activation contributes to the cognitive enhancement associated with increased IGF‐1 expression (Hu et al. 2016). However, in the present study, no significant differences were observed among the groups for phospho‐ERK and phospho‐ERK/total‐ERK ratio (Figure 6C).

In addition to IGF‐1, VEGF and fibroblast growth factor‐2 (FGF‐2) are also known to be involved in the improvement of cognitive function through exercise (Cao et al. 2004; Song et al. 2024; Jin et al. 2003; Tsuda 2025). We examined whether these molecules were significantly elevated in the exercise + L50 group, but neither the protein levels of VEGF nor FGF‐2 in the hippocampus differed significantly among the groups (Figure S2).

## 4 Discussion

Lactate, long considered to be an unnecessary metabolic byproduct, is now recognized as a crucial energy source and signaling molecule in numerous tissues. Our previous study revealed that combined intake of amino acid mixtures and exercise induces a significant increase in plasma lactate levels, thereby promoting the formation of beige adipocytes (Kojima et al. 2021). Furthermore, we found that oral intake of lactate alone induces the formation of beige adipocytes in white adipose tissue via reactive oxygen species production without exercise (Esaki et al. 2023). Building on these findings regarding the physiological roles of lactate, in the present study, we demonstrated that the chronic oral administration of lactate combined with voluntary wheel running significantly improved object and spatial recognition memory in young mice. Furthermore, we found that these cognitive‐enhancing effects were associated with the activation of the hippocampal IGF‐1/CaMKII signaling pathway.

Exercise is known to enhance energy metabolism and improve cognitive function. We previously reported that a combination of dietary factors and exercise synergistically induces the formation of beige adipocyte (Tsuda 2025). We also reported that the combined intake of a low dose of highly bioavailable curcumin formulation and exercise improves learning and memory abilities, whereas curcumin alone or exercise alone did not (Suzuki et al. 2025). These results suggest that instead of relying on exercise or dietary factors alone, dietary factors can be used as a substitute for exercise or together with exercise to amplify its beneficial effects (Tsuda 2025).

As mentioned above, lactate has been reported to improve cognitive function (Lev‐Vachnish et al. 2019). High‐intensity exercise increases blood lactate levels and enhances cognitive function (Rodriguez et al. 2018). However, maintaining a habit of high‐intensity exercise with the goal of improving cognition can be challenging. Therefore, combining lactate intake with exercise might effectively amplify the cognitive enhancement effects mediated by exercise or lactate.

In this study, we confirmed that high‐dose lactate intake (L100) significantly improves cognitive function, whereas low‐dose lactate intake (L50) alone does not. Based on these results, we hypothesized that combining exercise and L50 would effectively improve cognitive function while reducing the lactate dosage. We demonstrated that the combination of exercise and L50 significantly enhances learning and memory, whereas exercise alone does not. Notably, the combined intervention of exercise + L50 produced a remarkably large effect size (Cohen's *d* = 2.74 in NLR test), which exceeded the effect of high‐dose lactate alone (*d* = 1.43). The lack of a significant effect in cognitive performance in the exercise‐only group is consistent with our previous findings using the same 4‐week voluntary exercise protocol in young ICR mice (Suzuki et al. 2025). This suggests that the intensity or duration of voluntary wheel running in our experimental setting remained below the threshold required to independently elicit detectable cognitive enhancement in this mouse strain. In the hippocampus, only the exercise + L50 group showed a significant increase in the number of double‐positive BrdU/NeuN cells and expression of neural plasticity and synaptic activity markers. This reflects greatly enhanced neurogenesis in the exercise + L50 group. Therefore, the combination of exercise and L50 offers the advantage of significantly improving cognitive function without increasing lactate intake.

Next, we examined why the combination of exercise and L50 improves cognitive function. BDNF is a crucial molecule in cognitive enhancement, and exercise‐induced cognitive improvement is mediated by BDNF in the brain (Wrann et al. 2013). In the present study, exercise (with or without lactate) significantly increased hippocampal BDNF levels. However, while the exercise + L50 group showed improved cognitive function, the exercise alone group did not, despite having similar levels of BDNF. This suggests that the increase in BDNF alone was not sufficient to significantly enhance cognitive performance under our experimental conditions, and that other mechanisms must contribute to the synergistic effect observed in the combination group.

Therefore, the superior cognitive improvement observed in the exercise + L50 group cannot be fully explained by the upregulation of BDNF alone. Furthermore, we examined the relationship between individual BDNF levels and cognitive performance; however, no significant correlation was observed, likely due to individual variability and the limited sample size for protein analysis. This provides further evidence that the degree of BDNF elevation does not directly determine the extent of cognitive enhancement in our model. Although some studies have reported that lactate‐induced cognitive enhancement involves BDNF (El Hayek et al. 2019), our results showed that hippocampal BDNF protein expression was not significantly increased by lactate intake alone (L50 or L100) (Figure S1). These findings collectively suggest that while BDNF may contribute to the general response to exercise, other signaling pathways are likely responsible for the specific synergistic effect of exercise and lactate.

Other molecules expressed in the brain that mediate exercise‐related cognitive enhancement include VEGF, FGF‐2, and IGF‐1. VEGF is involved in increasing blood flow in the brain, thereby promoting neurogenesis and angiogenesis and enhancing spatial memory and learning (Cao et al. 2004). The induction of FGF‐2 also promotes cognitive function by increasing neurogenesis in the hippocampus (Song et al. 2024; Jin et al. 2003). IGF‐1 is expressed in the brain, and it enhances neuronal plasticity and neurogenesis in the brain (Glasper et al. 2010; Farias Quipildor et al. 2019). IGF‐1 plays a crucial role in the brain and is thought to contribute to enhanced cognitive function along with its downstream signaling pathways (Hu et al. 2016). In the present study, hippocampal VEGF and FGF‐2 protein expression did not show significant differences among the groups (Figure S2). In contrast, hippocampal IGF‐1 protein expression significantly increased only in the exercise + L50 group along with the induction of neurogenesis and the activation of CaMKII, which is an IGF‐1‐related downstream pathway, but not in the exercise‐alone group. In addition, 4 weeks of lactate administration alone did not significantly increase hippocampal IGF‐1 protein expression in either the L50 or L100 group (Figure S3). IGF‐1 is also expressed in the liver, a reflection of its plasma levels and involvement in cognitive function (Pharaoh et al. 2020). Additionally, IGF‐1 is expressed in skeletal muscle, and its expression increases with exercise (Matheny et al. 2009). In the present study, IGF‐1 protein expression did not significantly increase in the exercise + L50 group in the liver and skeletal muscle, and plasma IGF‐1 levels did not significantly differ among the groups. Therefore, the increase in hippocampal IGF‐1 protein expression in the exercise + L50 group was likely due to increased IGF‐1 expression in the hippocampus, contributing to the cognitive enhancement observed in the combination group.

This study has some limitations that warrant further consideration. First, we used young adulthood mice as an experimental model. While many studies focus on preventing age‐related cognitive decline, our objective was to explore the paradigm of cognitive enhancement in a healthy physiological state. Optimizing neuroplasticity during this stage is critical for long‐term brain health, and our findings provide a proof‐of‐concept that chronic lactate intake combined with exercise can enhance cognitive performance even in healthy individuals. However, the effects of this intervention on aged models or individuals with existing cognitive impairments remain to be elucidated. Second, regarding the delivery of lactate, oral intake may not lead to the high systemic concentrations typically observed during high‐intensity exercise. Nevertheless, our results suggest that chronic, moderate administration can exert beneficial effects through cumulative signaling pathways, offering a practical approach for daily health management. Specifically, this study did not examine whether similar effects could be achieved with reduced exercise intensity, a lower lactate dosage, or shorter experimental periods. Furthermore, while our findings strongly suggest the involvement of the IGF‐1/CaMKII signaling pathway in the observed cognitive improvements, this study does not provide direct causal evidence. Future research incorporating the use of specific inhibitors, such as an IGF‐1 receptor inhibitor, or gene‐silencing techniques would be necessary to further elucidate the definitive causal link between these molecular changes and the behavioral outcomes. Altering these variables in a future study would provide valuable data for utilizing lactate as a dietary supplement during exercise.

In conclusion, the combination of lactate intake and exercise improves learning and memory abilities, whereas exercise alone does not. This effect is likely related to increased IGF‐1 expression and the activation of CaMKII associated with IGF‐1 signaling in the hippocampus. Our results suggest that lactate can be used as a dietary supplement during exercise to enhance learning and memory abilities in both young and older individuals.

## Author Contributions

**Momoka Ota:** conceptualization, investigation. **Takanori Tsuda:** funding acquisition, conceptualization, writing – original draft, writing – review and editing, project administration. **Nana Esaki:** conceptualization, writing – original draft, investigation. **Kai Sahara:** investigation. **Toshiro Matsui:** conceptualization, writing – original draft, writing – review and editing, funding acquisition.

## Funding

This work was supported by Grants‐in‐Aid for Scientific Research from the Japan Society for Promotion of Science (JSPS KAKENHI) (23K05083, 21H04863).

## Conflicts of Interest

The authors declare no conflicts of interest.

## Supporting information


**Figure S1:** The protein expression of BDNF in hippocampus in the control, L50 and L100 groups. Protein levels are expressed as fold‐change relative to the control (= 1) after normalization to the GAPDH protein level. Data are presented as means ± SEM (*n* = 7). GAPDH, glyceraldehyde‐3‐phosphate dehydrogenase.
**Figure S2:** The protein expression of VEGF and FGF‐2 in hippocampus in the control, EX and EX + L50 groups. Protein levels are expressed as fold‐change relative to the control (= 1) after normalization to the GAPDH protein level. Data are presented as means ± SEM (*n* = 7). EX, exercise; GAPDH, glyceraldehyde‐3‐phosphate dehydrogenase.
**Figure S3:** The protein expression of IGF‐1 in hippocampus in the control, L50 and L100 groups. Protein levels are expressed as fold‐change relative to the control (= 1) after normalization to the GAPDH protein level. Data are presented as means ± SEM (*n* = 7). GAPDH, glyceraldehyde‐3‐phosphate dehydrogenase.
**Table S1:** Body weight and food intake in the control, L50 and L100 groups for 4 weeks.
**Table S2:** Body weight, food intake and total running distance in the control, EX and EX + L50 groups for 4 weeks.

## Data Availability

The data that support the findings of this study are available from the corresponding author upon reasonable request.
