# Supplementary material for: Combination of Voluntary Wheel Running and Oral Intake of Lactate Improves Object and Spatial Recognition Memory in Association With Hippocampal Insulin‐Like Growth Factor‐1 in Mice
Source: Food Sci Nutr. 2026 Jul 6;14(7):e72091. doi: 10.1002/fsn3.72091 (PMC13334461; doi:10.1002/fsn3.72091)
Supplement: Supplementary file 1 — Figure S1: The protein expression of BDNF in hippocampus in the control, L50 and L100 groups. Protein levels are expressed as fold‐change relative to the control (= 1) after normalization to the GAPDH protein level. Data are presented as means ± SEM (n = 7). GAPDH, glyceraldehyde‐3‐phosphate dehydrogenase. Figure S2: The protein expression of VEGF and FGF‐2 in hippocampus in the control, EX and EX + L50 groups. Protein levels are expressed as fold‐change relative to the control (= 1) after normalization to the GAPDH protein level. Data are presented as means ± SEM (n = 7). EX, exercise; GAPDH, glyceraldehyde‐3‐phosphate dehydrogenase. Figure S3: The protein expression of IGF‐1 in hippocampus in the control, L50 and L100 groups. Protein levels are expressed as fold‐change relative to the control (= 1) after normalization to the GAPDH protein level. Data are presented as means ± SEM (n = 7). GAPDH, glyceraldehyde‐3‐phosphate dehydrogenase. Table S1: Body weight and food intake in the control, L50 and L100 groups for 4 weeks. Table S2: Body weight, food intake and total running distance in the control, EX and EX + L50 groups for 4 weeks. [file FSN3-14-e72091-s001.pdf]

Supporting information

Combination of voluntary wheel running and oral intake of lactate improves object and spatial recognition memory in association with hippocampal insulin-like growth factor-1 in mice

Authors: Momoka Ota, Nana Esaki, Kai Sahara, Toshiro Matsui and Takanori Tsuda

1. Supporting materials and methods

The antibodies used in this study are shown in the tables below.

List of primary antibodies

Antibody	Manufacturer	Product No.
BDNF	Abcam, Tokyo, Japan	ab108319
PSD95	Cell Signaling Technology, Tokyo, Japan	#3409
Arc	Cell Signaling Technology, Tokyo, Japan	#65650
NeuN	Cell Signaling Technology, Tokyo, Japan	#24307
BrdU	Proteintech, Tokyo, Japan	66241-1-Ig
MCT2	Proteintech, Tokyo, Japan	20355-1-AP
IGF-1	Cell Signaling Technology, Tokyo, Japan	#73034
VEGF	Proteintech, Tokyo, Japan	81323-2-RR
FGF-2	Proteintech, Tokyo, Japan	11234-1-AP
p-IGF-1Rβ (Tyr1135)	Cell Signaling Technology, Tokyo, Japan	#3918
IGF-1Rβ	Cell Signaling Technology, Tokyo, Japan	#3027
p-CaNKII (Thr286)	Cell Signaling Technology, Tokyo, Japan	#12716
CaMKII	Cell Signaling Technology, Tokyo, Japan	#4436
p-ERK (Thr292/Tyr204)	Cell Signaling Technology, Tokyo, Japan	#9101
ERK	Cell Signaling Technology, Tokyo, Japan	#9102
GAPDH	Fujifilm Wako Pure Chemical Corporation, Osaka, Japan	016-25523

Immunoblot analysis. The tissue samples were homogenized, centrifuged and the total protein concentrations of the obtained supernatant were determined using a Protein Assay System (Bio-Rad, Richmond, CA) with bovine γ-globulin employed as a standard. Aliquots of

the supernatant were treated with Laemmli sample buffer for 5 min at 100 °C. The samples were then loaded onto an SDS-PAGE system. The resulting gel was transblotted onto a PVDF membrane, which was blocked with 5% skim milk for 1 h at room temperature. After a washing with 20 mM Tris-HCl-buffered saline containing 0.05% (w/v) Tween 20 (TTBS), the membrane sheets were reacted with various antibodies for 16 h at 4 °C. After a washing with TTBS, the membranes were reacted with horseradish peroxidase-conjugated anti-rabbit IgG secondary antibodies (1:2000 dilution; Cell Signaling Technology, Tokyo, Japan) for 1 h at room temperature. After a washing, immunoreactivity was visualized using the ECL reagent (Thermo Fisher Scientific, Yokohama, Japan), and the relative signal intensity was evaluated with iBright CL1500 Imaging System (Thermo Fisher Scientific).

2. Supporting Tables.

Table S1. Body weight and food intake in the control, L50 and L100 groups for 4 weeks.[1]

	Control	L50	L100
Initial body weight, *g*	37.1 ± 0.5	37.1 ± 0.5	37.1 ± 0.5
Final body weight, *g*	52.6 ± 2.2	52.6 ± 0.9	51.6 ± 1.3
Water intake, *g/(4 weeks ∙ mouse)*	136.8 ± 17.8	171.0 ± 12.5	179.5 ± 10.3
Food intake, *g/(4 weeks ∙ mouse)*	152.7 ± 6.1	148.2 ± 2.2	157.2 ± 3.7

[1] Values are presented as means ± SEM (n = 7).

Table S2. Body weight, food intake and total running distance in the control, EX and EX + L50 groups for 4 weeks.[1]

	Control	EX	EX + L50
**Initial body weight, *g***	**37.7 ± 0.5**	**37.8 ± 0.5**	**37.6 ± 0.5**
**Final body weight, *g***	**46.9 ± 1.5**[b]	**37.8 ± 1.3**[a]	**38.5 ± 1.7**[a2]
**Food intake, *g/(4 weeks · mouse)***	**137.6 ± 5.4**	**132.6 ± 3.0**	**132.4 ± 3.2**
**Water intake, *g/(4 weeks · mouse)***	**103.9 ± 4.6**[b]	**157.7 ± 7.5**[a]	**162.4 ± 12.9**[a]
**Running dstance, *km/(4 weeks · mouse)***	**-**	**264.8 ± 35.4**	**230.6 ± 28.5**

[1] **Values are presented as means ± SEM (*n* = 14).**

[2] **Values with different letters are significantly different ($p < 0.05$).**

EX, exercise.

3. Supporting Figures.

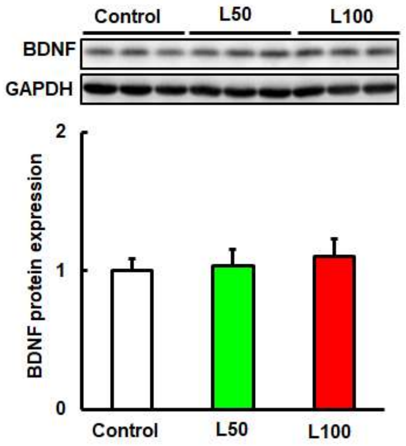

Figure S1. The protein expression of BDNF in hippocampus in the control, L50 and L100 groups. Protein levels are expressed as fold-change relative to the control (= 1) after normalization to the GAPDH protein level. Data are presented as means ± SEM (n = 7). GAPDH, glyceraldehyde-3-phosphate dehydrogenase.

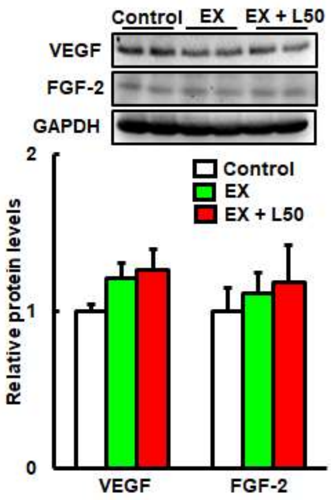

Figure S2. The protein expression of VEGF and FGF-2 in hippocampus in the control, EX and EX + L50 groups. Protein levels are expressed as fold-change relative to the control (= 1) after normalization to the GAPDH protein level. Data are presented as means ± SEM (n = 7).
EX, exercise; GAPDH, glyceraldehyde-3-phosphate dehydrogenase.

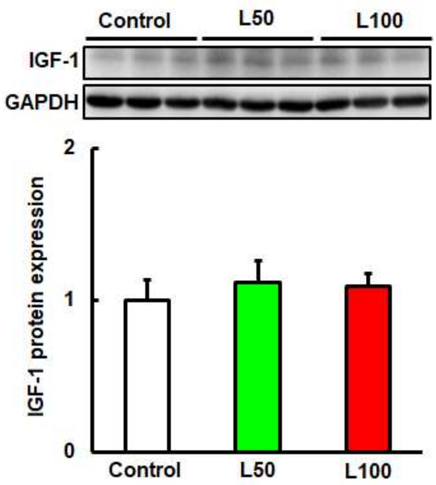

Figure S3. The protein expression of IGF-1 in hippocampus in the control, L50 and L100 groups. Protein levels are expressed as fold-change relative to the control (= 1) after normalization to the GAPDH protein level. Data are presented as means ± SEM (n = 7).
GAPDH, glyceraldehyde-3-phosphate dehydrogenase.
